# Biophysical Insight on the Membrane Insertion of an Arginine-Rich Cell-Penetrating Peptide

**DOI:** 10.3390/ijms20184441

**Published:** 2019-09-09

**Authors:** Marie-Lise Jobin, Lydie Vamparys, Romain Deniau, Axelle Grélard, Cameron D. Mackereth, Patrick F.J. Fuchs, Isabel D. Alves

**Affiliations:** 1 Institute of Chemistry & Biology of Membranes & Nanoobjects (CBMN), CNRS UMR5248, University of Bordeaux, Bordeaux INP, allée Geoffroy St-Hilaire, 33600 Pessac, France; 2 University of Paris, Institut Jacques Monod, CNRS, 75013 Paris, France; 3 ARNA Laboratory, INSERM U1212, CNRS UMR5320, University of Bordeaux, IECB, 2 rue Robert Escarpit, 33600 Pessac, France; 4 Laboratoire des biomolécules (LBM), CNRS UMR7203, Sorbonne University, École normale supérieure, PSL University, 75005 Paris, France; 5 University of Paris, UFR Sciences du Vivant, 75013 Paris, France

**Keywords:** cell-penetrating peptide, peptide–lipid interaction, lipid model systems, molecular dynamics, NMR, membrane biophysics

## Abstract

Cell-penetrating peptides (CPPs) are short peptides that can translocate and transport cargoes into the intracellular milieu by crossing biological membranes. The mode of interaction and internalization of cell-penetrating peptides has long been controversial. While their interaction with anionic membranes is quite well understood, the insertion and behavior of CPPs in zwitterionic membranes, a major lipid component of eukaryotic cell membranes, is poorly studied. Herein, we investigated the membrane insertion of RW16 into zwitterionic membranes, a versatile CPP that also presents antibacterial and antitumor activities. Using complementary approaches, including NMR spectroscopy, fluorescence spectroscopy, circular dichroism, and molecular dynamic simulations, we determined the high-resolution structure of RW16 and measured its membrane insertion and orientation properties into zwitterionic membranes. Altogether, these results contribute to explaining the versatile properties of this peptide toward zwitterionic lipids.

## 1. Introduction

The biological membrane is one of the key structural elements of living cells, and constitutes the first barrier that is encountered by molecules and ions that need to be transported into cells. The ability to deliver drugs to the interior of cells is critical for diagnosis and therapeutic applications, and cell-penetrating peptides (CPPs) can overcome this limitation [1]. CPPs constitute a heterogeneous class of small peptides that can translocate through cell membranes and transport cargoes into cells, in a receptor- and energy-independent process. A major advantage of CPPs is their passivity towards cells, i.e., they do not present cytotoxicity. Their mode of action has been considerably studied since their discovery in the 1990s (for a detailed review, see [2]) and it is currently accepted that several parameters in their primary sequence are essential to confer these properties, including a net positive charge (high arginine or sometimes lysine content) and an optimal balance between charged amino acids and hydrophobic ones, i.e., amphipathcity [3,4,5]. These properties are often shared with antimicrobial peptides (AMPs), whose main role is to kill bacteria, but it is not clear how these similar peptides can exert extremely different functions. Altogether, these membrane-active peptides (MAPs) exert their biological activity by initially interacting with the plasma membrane, and therefore investigating the peptide-membrane interaction of such molecules is essential to understand their mode of action.

As plasma membranes are mainly composed of lipids, the peptide–lipid interactions are crucial for the initial binding of CPPs prior to internalization [6,7,8]. Polar residues, and especially arginines (Arg), interact with high affinity through their guanidinium group to negatively-charged lipids and lipid phosphate groups, and thus enhance the binding of Arg-rich peptides to membranes [9,10]. In parallel, hydrophobic residues such as tryptophans (Trp) have been shown to establish hydrophobic contacts with lipid acyl chains and play a role on the insertion of MAPs into the membrane [11,12]. Structural plasticity of these peptides during membrane contact may then bring sufficient peptide charge neutralization (e.g., through electrostatic interactions) to help the peptides translocate. However, it has also been suggested that highly hydrophobic residues might prevent peptide internalization, with the peptide trapped in the membrane due to these strong interactions [7,13]. Although CPPs have been broadly reported to have an enhanced affinity for negatively-charged membranes, peptide interaction and insertion in zwitterionic membranes is not fully described. A large amount of studies is performed on anionic systems (negatively-charged membranes) due to the establishment of important electrostatic interactions between the CPP and the cell membrane that are important for their internalization. While lipids in the outer eukaryotic cell membrane leaflet are mainly zwitterionic, with less than 2% anionic lipids, the cell membrane possesses an anionic character due to the glycosaminoglycans. Therefore, study of CPP interaction with zwitterionic lipids is important and most of the studies, to the best of our knowledge, mainly use zwitterionic systems as a comparison model to anionic ones.

In this study, we investigated one CPP, RW16 (RRWRRWWRRWWRRWRR), which possesses cell internalization capacity, but also shows antimicrobial and antitumor activity [14,15]. The design of RW16 was derived through structure–activity relationship (SAR) studies from penetratin (pAntp), and is composed of 10 Arg and 6 Trp to form an idealized amphipathic peptide. RW16 has been successfully shown to be an efficient CPP while exhibiting no cytotoxicity on fibroblast cells [15,16].

The interaction of RW16 with anionic or zwitterionic membranes has been fully described, but reveals atypical and poorly understood behaviors when interacting with zwitterionic liposomes [14,17]. Only a few studies to date have investigated this behavior at the molecular level. Lamazière et al. have shown that RW16 induced giant unilamellar vesicles (GUVs) via adhesion and aggregation with anionic lipid membrane compositions [17]. They also found that RW16 induced calcein leakage from liposomes, which suggests a link to membrane perturbation but remains non-lethal to cells at comparable concentrations. In a previous study, we observed that RW16 possesses enhanced membrane interaction and perturbation of membranes containing anionic lipids. This property could, in part, explain its antitumor and antibacterial activity, as cancer cells and bacteria contain anionic lipids in the outer leaflet of their membranes [14]. In contrast, RW16 shows weak perturbation of zwitterionic membranes, although this interaction is associated with fast and strong calcein leakage [14]. The polyvalent property displayed by RW16 is not observed for other CPPs and, therefore, it is important to understand it at a molecular level.

Herein, we focus on the first stages of interaction and membrane insertion of RW16 in contact with zwitterionic membranes (i.e., mimicking “healthy” cell membranes). By applying complementary and multidisciplinary biophysical methods, we investigated the molecular behavior of the peptide in contact with zwitterionic liposomes. Using nuclear magnetic resonance (NMR) spectroscopy, we calculated the structure of RW16 in the presence of zwitterionic micelles and used the NMR structure coordinates to perform molecular dynamics (MD) simulations. This approach allowed us to provide a complete molecular view of the peptide structure and orientation while inserted in the membrane, including insertion depth in zwitterionic membranes. We also provide an explanation for its ambiguous effect previously observed on zwitterionic membranes in comparison to anionic membranes.

## 2. Results

### 2.1. Ensemble Insertion Analysis of RW16 vs. Penetratin in Zwitterionic Membranes

**Figure 1:**
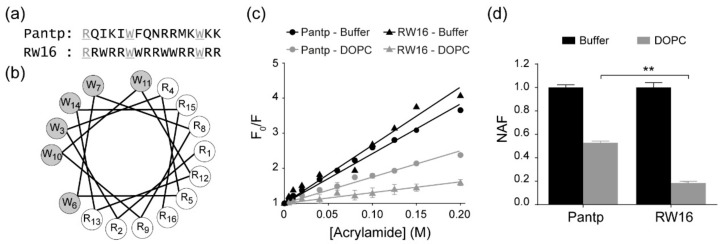
RW16 and penetratin insertion in the membrane. (**a**) Sequence alignment of RW16 and penetratin. (**b**) Edmunson wheel of RW16 along the axis generated from Helixator, http://www.tcdb.org. (**c**) Inhibition rate (F_0_/F) of RW16 and penetratin Trp fluorescence in buffer and in the presence of DOPC liposomes (P/L 1:50 mol:mol), with increasing concentrations of acrylamide. (**d**) Normalized accessibility factor (NAF) for penetratin and RW16 in presence of DOPC liposomes. Significance was tested with a Student’s *t*-test, where ** 0.001 < *p* < 0.01.

RW16 is a cell-penetrating peptide (CPP) derived from the well-characterized penetratin peptide (pAntp), and our initial approach was to compare RW16 to penetratin by studying membrane insertion into liposomes (LUVs, large unilamellar vesicles). To this aim, we employed the neutral hydrophilic quencher acrylamide to quench intrinsic Trp fluorescence of the peptides. Acrylamide is unable to penetrate the hydrophobic membrane core, such that only fluorophores not embedded in the bilayer are quenched. However, it should also be noted that the response is an ensemble average response from all Trp residues in the peptide sequence. Tryptophan quenching experiments of penetratin have been previously performed by other groups using different experimental conditions (e.g., different buffer, P/L ratio) [18,19]. It was shown that Trp residues locate at the interface between the polar headgroup region and the hydrophobic core of the lipids [6,20]. Using a Trp fluorescence quenching approach, we measured insertion properties of RW16 and penetratin into a zwitterionic membrane. Fluorescence spectra of the peptides were recorded in the absence and presence of liposomes of dioleoylphosphatidylcholine (DOPC), and with increasing concentrations of acrylamide. Stern–Volmer plots of acrylamide quenching are shown in Figure 1c. In buffer, the Stern–Volmer constant (K_SV_) of the two peptides were similar and comparable to the values found in the literature for similar concentrations of peptide and under equivalent experimental conditions [19,21]. In the presence of LUVs composed of DOPC, the Stern–Volmer constant of both peptides decreased significantly, demonstrating a strong insertion of the peptides in the membrane. The Stern–Volmer coefficients (K_SV_) were normalized to the K_SV_ calculated in buffer (NAF for “normalized accessibility factor”) to allow for the comparison between both peptides (Table 1 and Figure 1d) [11,21]. A higher NAF value corresponds to higher Trp exposure to solvent, and inversely, a lower NAF value relates to higher insertion in the bilayer. The NAF of RW16 in DOPC was lower than for penetratin (0.18 compared to 0.53; Table 1), which highlights that RW16 is inserted deeper in the membrane than penetratin. Based on the composition of RW16, the higher number of Trp as compared to penetratin might explain this finding, since additional Trp residues are predicted to have stronger hydrophobic contacts with the hydrophobic core of the membrane.

### 2.2. Solution Structure of RW16 in the Presence of Zwitterionic Membranes

**Figure 2:**
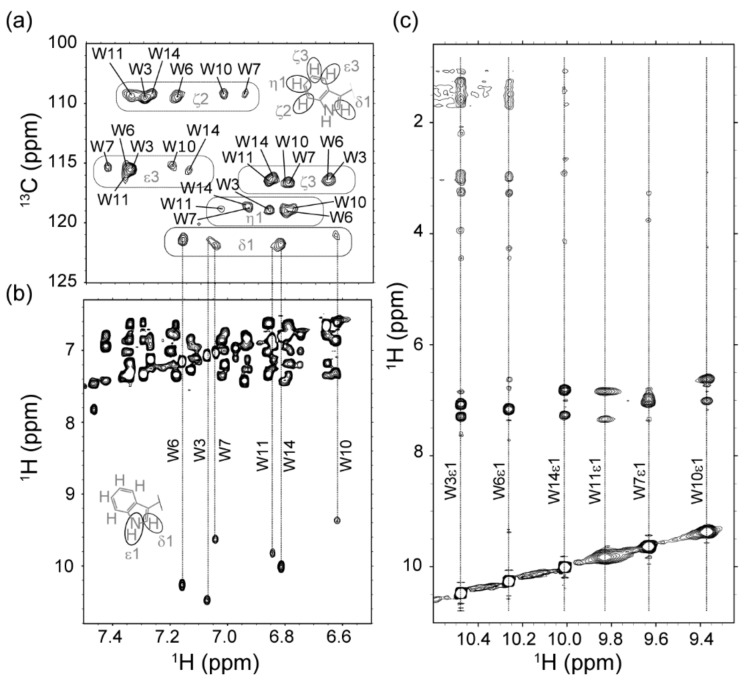
NMR spectroscopy of RW16 in DPC-d_38_ micelles. (**a**–**c**) Chemical shift assignments of RW16 using a combination of 2D ^1^H,^13^C-HSQC, 2D ^1^H,^1^H-TOCSY, and 2D ^1^H,^1^H-NOESY at 310 K. (**a**) Selected region of 2D ^1^H,^13^C-HSQC, illustrating assigned δ1, ζ2, η2, ε3, and ζ3 ^1^H-^13^C resonances for the 6 Trp residues. (**b**) Selected region of the 2D ^1^H,^1^H-TOCSY, highlighting assignment of the Trp ε1 ^1^H resonances from the δ1 crosspeaks in the 2D ^1^H,^13^C-HSQC. (**c**) Selected regions from the 2D ^1^H,^1^H-NOESY spectrum used to obtain distances for structure calculation, with representative NOE strips indicated for Trp ε1 ^1^H nuclei.

**Figure 3:**
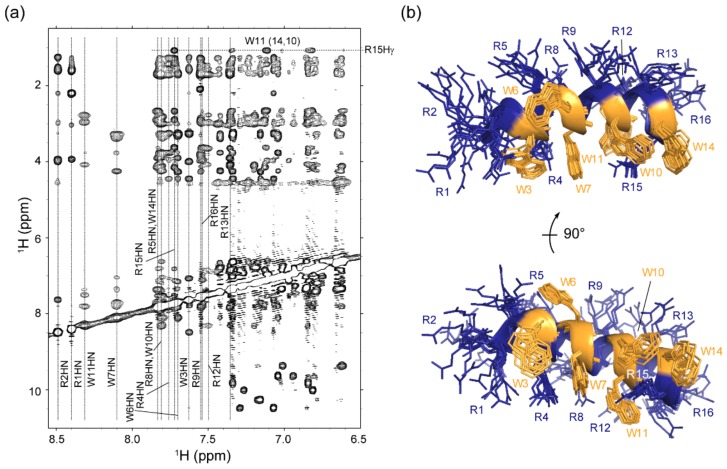
Solution structure of micelle-bound RW16. (**a**) Selected regions from the 2D ^1^H,^1^H-NOESY spectrum used to obtain distances for structure calculation, with representative NOE strips indicated for all backbone amide ^1^H^N^ nuclei. The upfield shifted side chain ^1^Hγ resonance of Arg15 is also indicated, with NOE crosspeaks to Trp11 and Trp14. (**b**) Ensemble of 10 structures calculated for RW16 bound to DPC-d_38_ micelles. The 6 Trp residues (orange) and 10 Arg residues (blue) are labeled. Note that the N-terminal biotin-aminopentanoic acid tag, although present in the sample, was not included in the structural models. The ensemble of structures has been deposited in the Protein Data Bank under accession number 6RQS.

To further address details of membrane insertion and to obtain information on RW16 at the atomic level, we next calculated the structure of the peptide in the presence of zwitterionic lipids. We prepared a sample of RW16 with dodecylphosphocholine-d_38_ (DPC-d_38_) micelles and used NMR spectroscopy to obtain distance restraints for structural calculation. DPC was employed instead of DOPC to obtain the high-resolution structure of the peptide, which would not be possible with DOPC liposomes using liquid-state NMR spectroscopy. The use of deuterated lipids in the sample allowed us to remove lipid contributions from the NMR signal and thus only observe peptide resonances. Strikingly, and as illustrated in Figure 2, the crosspeak signals in natural abundance 2D ^1^H,^13^C-HSQC (Figure 2a), 2D ^1^H,^1^H-TOCSY (Figure 2b), and 2D ^1^H,^1^H-NOESY (Figure 2c) were very well resolved and separated, despite the peptide sequence symmetry and only two types of residues (Arg and Trp). All ^1^H chemical shifts for the 6 Trp can be unambiguously assigned, as can the ^1^H resonances for the 10 Arg. The chemical shift assignments have been deposited in the Biological Magnetic Resonance Bank (BMRB) under accession number 34400. The 2D ^1^H,^1^H-NOESY (Figure 2c and Figure 3a) is indicative of a single population of structures, and we were able to derive 408 distance and 28 backbone dihedral restraints for structure calculations using Aria1.2/CNS1.21 [22] (Table 2). The ensemble of the 10 lowest energy structures of RW16 in the presence of DPC micelles is presented in Figure 3b and has been deposited in the PDB (Protein Data Bank) as entry 6RQS. As predicted, we observe that the Arg and Trp are mainly segregated to each side of the helix, thus creating an amphipathic helix. Arg15 appears to be an exception and seems to be isolated from the others as it is located on the “Trp side” of the helix. This arginine has upfield shifted chemical shift values and displays nuclear Overhauser effect (NOE) crosspeaks to neighboring Trp side chains (Figure 3a). Although not included in the structure calculation, the peptide also contains a N-terminal biotin-aminopentanoic acid tag (Biot-Apa).

**Figure 4:**
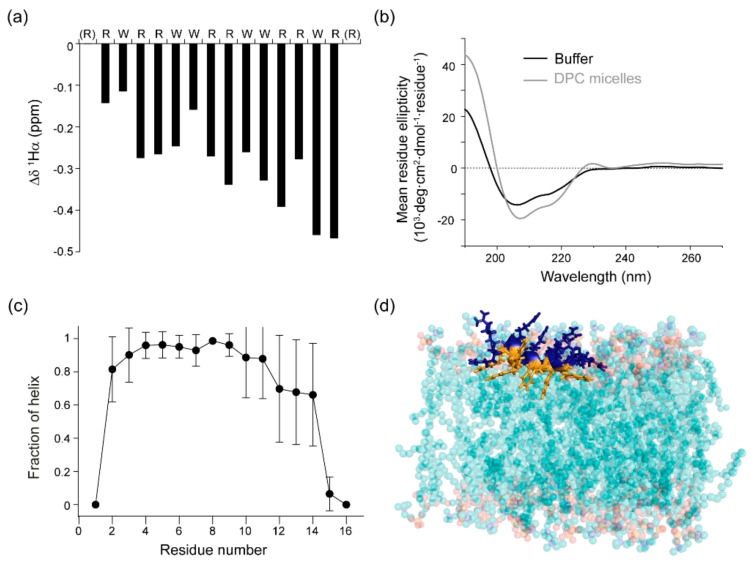
RW16 peptide structure in contact with zwitterionic membranes. (**a**) ^1^Hα chemical shifts compared to random coil ^1^Hα predictions obtained from NMR data. (**b**) CD spectra of RW16 in phosphate buffer (black line) and in the presence of DPC micelles (gray line). (**c**) Fraction of helix calculated from the molecular dynamics (MD) simulations. The three states H (α-helix), G (3_10_ helix), and I (π-helix) of the DSSP program were considered as part of the helix fraction (see Materials and Methods). After discarding the first 10 ns, each trajectory was cut into two blocks. Each value ± error was calculated as the mean and standard deviation over the six blocks respectively. (**d**) Snapshot of RW16 inserted in DOPC bilayer at *t* = 633 ns of MD trajectory 1. The C-terminus is on the left and N-terminus on the right. Trp are represented in orange, Arg in blue, the backbone is shown as an orange/blue ribbon and the lipids are drawn as spheres where carbon atoms are in cyan, oxygen in red, and nitrogen in blue.

A notable feature of the micelle-bound RW16 peptide is the high degree of helicity. The helical αnature of bound RW16 is supported both by NOE crosspeaks (Figure 2 and Figure 3a), as well as the negative secondary chemical shift values of the backbone ^1^Hα nuclei (Figure 4a). To confirm this observation, we measured the secondary structure content of RW16 by circular dichroism (CD) in buffer and in the presence of DPC micelles (Figure 4b). By deconvoluting the measured CD signal, we observe a predominant α-helical structure for the peptide in buffer, as well as in the presence of zwitterionic lipids (Table 3). We also measure an increase in the α-helix content with zwitterionic membranes compared to buffer. Furthermore, the helical content is slightly higher (76% vs. 60%) with micelles than with LUVs. This trend was previously found in Jobin et al. [14] and demonstrates a stronger structuring of the peptide in presence of micelles (Table 3).

Molecular details of membrane interaction with RW16 could not easily be obtained from our NMR spectroscopy data, due to the necessary use of deuterated lipids such that NOEs between peptide and lipids were not visible. Therefore, we decided to use molecular dynamics (MD) simulations in a zwitterionic membrane, using the solution structure of membrane-bound RW16 as a starting point in the simulations. This method allowed us to get insight into the burying of RW16 in DOPC membranes on a microsecond time scale (Supplementary Movie 1).

Our first analysis of the MD simulations confirmed a persistent helical structure of RW16, with the helix fraction calculated over the three trajectories shown in Figure 4c and a snapshot is shown in Figure 4d (Supplementary Movie 1). The segments 2–9 display a very stable helix fraction (80 to 100%). The segments 10–14 also remain helical but are more labile with some excursions to a turn (T state), explaining the larger error bars. Interestingly, we also observed these segments fluctuating between some turn state and π-helix in the first trajectory (Figure S1). The occurrence of a π-helix corresponds to a “compression” of the helix on these somehow short segments of the peptide. Overall, our MD trajectories show that the peptide remains helical with some possible fluctuations in the backbone hydrogen bonds on the C-terminal part.

Consistent with our NMR data, we observed very small fluctuations, confirming a strong anchoring of RW16 to the membrane thanks to its Trp side chains. Figure 4d shows a snapshot of the peptide being inserted in the membrane of DOPC after 633 ns of one simulation (Supplementary Movie 1). Visual observation of the MD trajectories in the DOPC membrane, moreover, revealed that the azimuthal angle of RW16 (how the peptide rotates about its helix axis) is very constant with the face of Trp residues oriented towards the membrane center and the Arg residues towards the interface membrane/water. These findings, however, also differ from the NMR results, in which a more flexible structure is observed on the N-terminal side (Figure 4a). This variation can arise from different parameters and will be further detailed in the Discussion.

### 2.3. Membrane Insertion Depth of RW16

**Figure 5:**
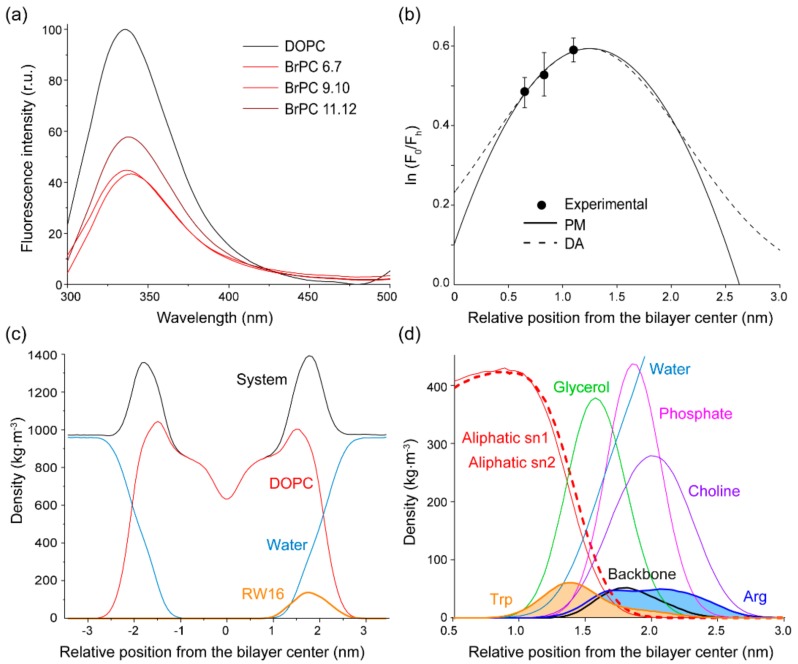
Peptide insertion into the membrane. (**a**) Representative Trp fluorescence spectra of RW16 in the presence of DOPC or DOPC/BrPC liposomes. (**b**) Curve fitting calculated by distribution analysis (DA) or the parallax method (PM) for RW16 in zwitterionic vesicles. The data were averaged over four independent experiments and each value ± error represents the mean and standard deviation. (**c**) Density profiles along the perpendicular axis to the bilayer plane calculated by MD simulations corresponding to water molecules, DOPC molecules, peptides, and the overall system. (**d**) Close up from (c) on the Trp and Arg region of the peptide, and on the lipid subgroups.

To obtain more precise information regarding the membrane partitioning of RW16, and more specifically side chain insertion depth in the lipid bilayer, quenching of Trp fluorescence by brominated lipids was performed. Three different lipids, each containing two bromines covalently attached to the lipid acyl chains at three different positions, were incorporated in DOPC LUVs. These lipids act as an internal probe in the liposome membranes with a quenching radius of 8–9 Å for the brominated probes [24]. It was previously shown that the presence of two bromines on the lipid acyl chain does not modify the physical properties of the lipids (like the phase transition) and preserve the lipid packing properties as in DOPC lipids [25]. Therefore, these are unlikely to influence the peptide-membrane interaction. Trp fluorescence of RW16 was measured in the presence of liposomes composed of DOPC alone or DOPC with a small percentage (30% mol:mol) of the brominated lipids (Figure 5a). The quenching observed in the presence of the different liposomes was normalized to the fluorescence measured in pure DOPC LUVs. The quenching percentage provides the average distribution of Trp from the bilayer center using equations of the distribution analysis (DA) and of the parallax method (PM) (for more details, see Materials and Methods) [21,26,27,28,29,30] (Figure 5b). Our calculation indicates that Trp residues are, on average, inserted at 12–13 Å from the bilayer center (Table 4). According to Wiener et al. [31], DOPC bilayers have a hydrocarbon core of approximately 30 Å and a total bilayer thickness of around 50 Å. We therefore determined the peptide to be located at around 7 Å from the polar headgroup of lipids, at the interface between the hydrophobic core and the polar region. The broad area calculated for Trp insertion depth might arise from different bilayer environments due to heterogeneity of the different Trp residue locations in the bilayer, also indicating that the helix might be tilted. In comparison, penetratin was observed to be located at the glycerol and phosphate levels upon zwitterionic bilayer insertion, but to a lesser extent than observed for RW16 [32,33,34]. These results, together with the acrylamide quenching experiments, confirm that RW16 inserts stably into zwitterionic bilayers. In addition, these results could explain, at least partially, our previous observation of calcein leakage measured in zwitterionic lipids and the membrane perturbation of zwitterionic vesicles [14]. In the case of anionic membranes, the interaction between Arg and lipid headgroups creates a charge compensation which delays calcein leakage, whereas the net charge of 0 at the membrane surface of zwitterionic liposomes creates an imbalance in the peptide-lipid interaction and higher fluctuations of the membrane [6].

Figure 5c shows the average location of RW16 in DOPC bilayer, calculated from MD simulations at 17 Å from the bilayer center, which is just below the polar headgroups of the lipids. A detailed analysis of the side chain location allowed us to precisely calculate an average insertion depth of Trp residues to be 13 Å from the bilayer center (Figure 5d and Figure S2). This is in agreement with fluorescence spectroscopy results and confirms that Trp residues are located adjacent to the phospholipid glycerols at the interface between the polar and the hydrophobic region of lipids. This placement is not surprising, given the preference of Trp residues for interfacial partitioning [12,35]. In parallel, Arg residues displayed a broad location between 17 and 22 Å from the bilayer center (Figure 5d and Figure S2), suggesting the presence of electrostatic interactions formed by bidentate hydrogen bonds of Arg and negative charges of the phosphate of the DOPC.

The peptide insertion is further illustrated by following the relative exposure of the center of mass (COM) of RW16 side chains in comparison to the lipid atoms (Figure S2). We observed that Trp3, Trp6, Trp7, and Trp10 are buried in the hydrophobic part of the bilayer and located below the central glycerol atom (around 13–14 Å from the bilayer center). In contrast, Trp11 lies at the phosphate level and Trp14 lies at the glycerol level, being therefore both slightly exposed to the solvent. The naf value observed with acrylamide quenching experiments for RW16 might be due to these two amino acids still being exposed to the solvent. As expected, Arg residues are mostly located between the central glycerol atom and the nitrogen of the choline. More specifically, Arg1, Arg2, Arg4, Arg13, and Arg15 lie between the phosphate and the glycerol and are therefore less solvent-exposed. Arg5, Arg8, Arg9, Arg12, and Arg16 are located above the choline nitrogen and are therefore highly solvent-exposed. The total number of hydrogen bonds between the Arg side chains and lipids were further calculated for the three trajectories from MD simulations (Figure S3). We observed an increase in the number of hydrogen bonds during the simulations, starting from 14 (±1, SD) hydrogen bonds reaching a plateau after 500 ns where 19 (±2, SD) hydrogen bonds were calculated (Figure S3a). This correlates well with the presence of 10 bidentate bonds occurring between all 10 Arg side chains and the lipid phosphate groups. Moreover, we calculated the number of Arg–water hydrogen bonds (Figure S3b), and found that they instead slightly decrease from 30 (±2, SD) hydrogen bonds and tend to a plateau at 27 (±2, SD) after a few hundreds of ns. This could indicate the presence of 10 Arg–water hydrogen bonds at the beginning of the simulation and suggests that one Arg might form different types of bonds during the simulations. This is in agreement with our previous results that showed Arg15 underwent important changes in its partitioning during one simulation and the observed shifted chemical shift values and NOE crosspeaks to neighboring Trp side chains observed in NMR.

Overall, the results on solvent accessibility by MD are similar to an insertion of the Trp at around 13 Å from the bilayer center, revealed by Trp fluorescence quenching by acrylamide. This insertion trend is in line with the fact that the peptide stabilizes into the bilayer by creating hydrophobic interactions with the fatty acid chains through Trp residues and electrostatic interactions with the lipid phosphates through Arg residues. A similar behavior has been reported for other cell-penetrating peptides [18,21].

### 2.4. Tilting of RW16 in the Membrane

**Figure 6:**
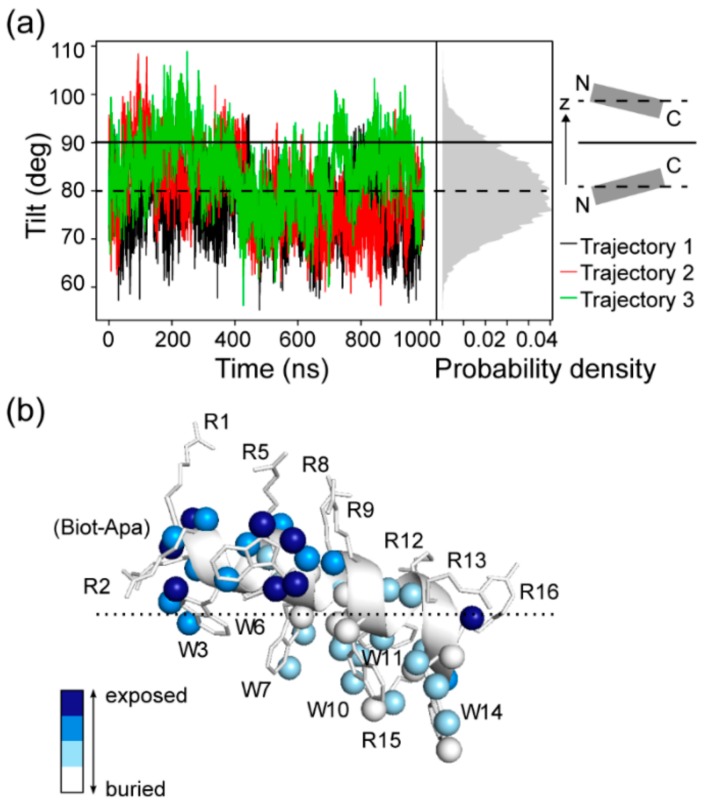
Peptide tilting relative to the normal to the zwitterionic membrane plane calculated by NMR and MD. (**a**) Tilt of the peptide calculated from the difference in z position between the C-alpha of Arg2 and Arg13 calculated over the three trajectories. The histogram (gray) shows the average distribution of z over the three trajectories. The scheme on the right panel shows that a tilt <90° describes a deeper insertion of the N-terminus, while a tilt >90° describes a deeper insertion of the C-terminus. (**b**) Solvent accessibility for RW16 in DPC micelles, as measured by NMR spectroscopy using the paramagnetic probe Gd(DTPA-BMA). The resulting solvent paramagnetic relaxation enhancement values (sPRE; Figure S4) have been measured for several atoms in RW16 and shown as spheres. Atoms that are strongly affected (colored blue) by the added Gd(DTPA-BMA) are more solvent-exposed as compared to atoms that are less affected (colored white). The tilt of the peptide shown represents an estimate based on the observed data.

We have demonstrated that RW16 is not fully inserted in the bilayer, with some exposure to the aqueous buffer through Arg residues. Previously, Walrant et al. observed that some CPPs are tilted in the bilayer [9], and thus we decided to investigate peptide tilting of RW16 relative to the normal to the bilayer plane. Using the MD simulation data, we were able to precisely calculate a tilt angle of the peptide, defined as the angle between the helix and the normal to the bilayer plane (Figure 6a). Because of the perfectly amphipathic nature of RW16 (Figure 1b), an interfacial partitioning with a horizontal orientation (i.e., tilt of 90°) within the membrane/water interface is energetically more favorable than any low tilt angle [36]. However, we noted that the peptide is not perfectly horizontal in the MD trajectories and presents a slight tilt, with values ranging from 75° to 85°, with an average tilt value around 80°. These results indicate that the N-terminus of the peptide is inserted deeper in the membrane. This trend is consistent with the insertion depth calculations performed for each residue (Figure 5d and Figure S2), with the N-terminal side chains systematically more buried inside the bilayer compared to the C-terminal side chains. Specifically, the Cα of Arg1 is located between the phosphates and the glycerol atoms, whereas the Cα of Arg16 is located above the cholines.

Similar to our results, it was shown that Trp residues of penetratin are facing towards the hydrophobic core of the bilayer [34]. In addition, the results on penetratin show a similar tilting behavior of the peptide. For penetratin, the Trp residues are located at the level of phospholipid glycerols (in a zwitterionic bilayer) with the N-terminus of the amphipathic helix inserted slightly deeper into the bilayer than the C-terminus (tilt angle of 80 to 90°) [20,33]. The small difference in tilt angles between the two peptides may arise from the high density of Trp in RW16, which could induce stronger anchoring of Trp residues to the lipid acyl chains in the hydrophobic core of the membrane.

The orientation of RW16 was also investigated by solution NMR spectroscopy with the sample prepared in the zwitterionic membrane-mimicking DPC micelles. The peptide position in micelles was examined by measuring the accessibility of atoms to the solvent (buffer) as a function of increasing concentrations of the paramagnetic agent Gd(DPTA-BMA). This paramagnetic probe is water-soluble and inert toward peptide-micelle complexes, and leads to faster relaxation towards nuclei in a distance-dependent manner [37]. Therefore, the highest solvent paramagnetic relaxation enhancement (sPRE) is expected for solvent-exposed residues outside of the bilayer, followed by residues at the water/bilayer interface. The sPRE values were measured for several atoms (Figure S4) and the results mapped to the RW16 peptide (Figure 6b). The pattern of accessible atoms reflects a situation in which the N-terminus is more exposed to the buffer as compared to the C-terminus, and therefore indicates a tilt angle greater than 90°. This behavior is opposite to the main tilt observed in the MD simulations. It is likely that this difference arises from the nature of the N-termini: In the NMR spectroscopy measurements, a biotin is linked by the Apa spacer to the N-terminus, whereas a simple acetyl group is used in the MD simulations. The larger biotin is hydrophilic and is not expected to insert deeply into the hydrophobic core of the membrane. In keeping with this hypothesis, the biotin nuclei are highly sensitive to the addition of Gd (DPTA-BMA) (Figure S4) and are therefore exposed to the buffer. The altered tilt preference in the NMR sample may also help explain the fact that RW16 displays a dynamic N-terminus, whereas the N-terminus was structured in the MD simulations and the C-terminus was more labile (with the helix undergoing more fluctuations). Nevertheless, we find by both methods that one terminal side is less partitioned into the membrane, and this side is more flexible than the rest of the helix.

### 2.5. Side Chain Contacts of RW16 in the Bilayer

**Figure 7:**
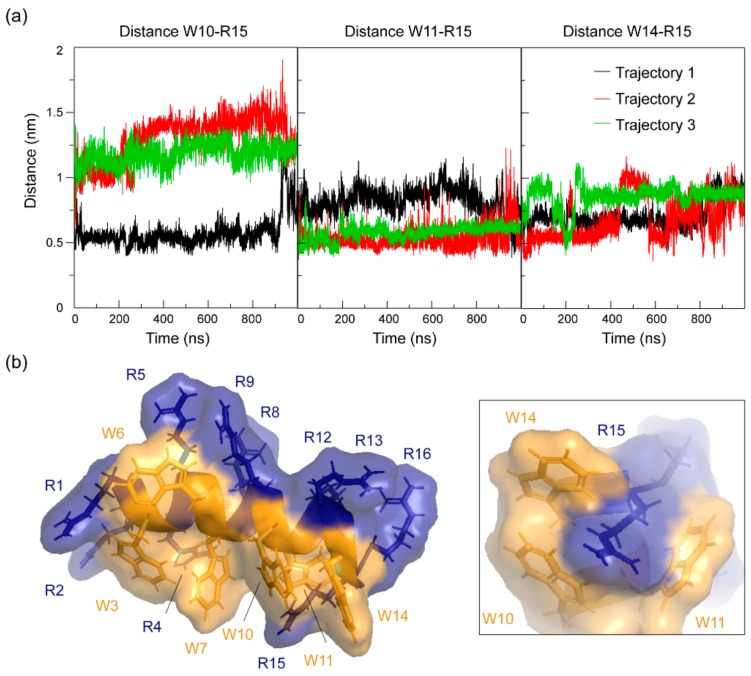
Side chain contacts between Arg15 and residues Trp10, Trp11, and Trp14. (**a**) COM-COM of side chain distances between Trp10 and Arg15 (left, W10–R15), Trp11 and Arg15 (middle, W11–R15), and Trp14 and Arg15 (right, W14–R15) for the three trajectories. (**b**) Surface density of RW16 amino acids showing a special arrangement of Arg15 and Arg–Trp π-cation interactions (left). Close-up on the pocket formed by Trp10, Trp11, and Trp14 around Arg15 (right). Structure generated with Pymol (PDB ID: 6RQS) [38].

As previously shown by NMR spectroscopy, MD simulations, and fluorescence spectroscopy, the Trp residues in RW16 are strongly anchored in the membrane, which leads to a high stability of the peptide in the bilayer. In contrast, most of the Arg residues extend out of the membrane, with the sole exception of Arg15. As already mentioned, we observed chemical shift values for the Arg15 side chain that are upfield shifted in keeping with a more hydrophobic environment, and we observed clear NOE crosspeaks to side chain atoms of Trp11, as well as Trp10 and Trp14 (Figure 3a). Calculations of the distance between residues in MD simulations also revealed side chain–side chain contacts, mainly between Arg15 and Trp11, but also with Trp10 and Trp14 (Figure 7a). This is consistent with what is observed in NMR and suggests that Arg15 is in a dynamic cavity surrounded by three Trp residues and creates π-cation contacts with them (Figure 7b). The contact to Arg15 may also explain the line-broadening of several nuclei from Trp11, such as the exchangeable side chain hydrogen ^1^Hε (Figure 2c). Similar Arg–Trp π-cation contacts were also observed for penetratin and were suggested to help stabilize the peptide inside the bilayer by partially masking its positive charge [33].

## 3. Discussion

**Figure 8:**
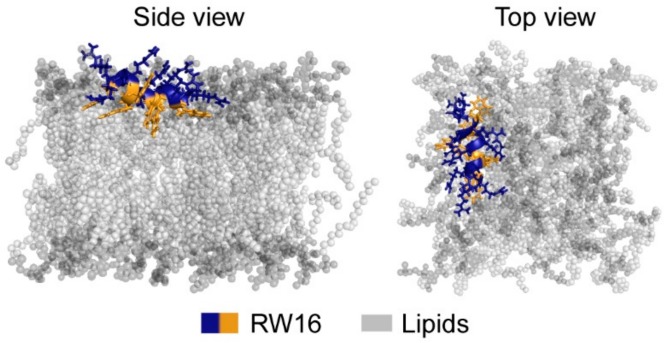
Summary cartoon representing RW16 embedded in a zwitterionic membrane. Shown is the calculated secondary structure and orientation of the peptide with the Trp (orange) and Arg (blue) side chains in a zwitterionic membrane (gray) with the polar headgroups (dark gray) and the aliphatic chains (light gray).

By using a multidisciplinary approach, we have determined the structure and membrane insertion of RW16, an amphipathic and cationic CPP, in the presence of zwitterionic bilayers. Previous data on RW16 highlighted its versatile properties in terms of biological activity and membrane interaction [14,15,16,17,39]. While the enhanced interaction and perturbation of anionic membranes by CPPs have been extensively studied, herein we provide a detailed molecular view of the membrane insertion of RW16 into zwitterionic membranes summarized in Figure 8.

We show that RW16 conformation is mostly α-helical in buffer, and this helicity is slightly enhanced in the presence of zwitterionic liposomes. The use of different membrane models (micelles and LUVs) did not generate significant differences in the secondary structure of the peptide. This is similar to previous observations made on other CPPs [40].

Using fluorescence spectroscopy, NMR, and MD methods, we obtained precise side chain insertion values, i.e., at the interface between the polar and the hydrophobic region of lipids (Figure 8). These values are comparable to calculated insertion depths of penetratin, the parent CPP of RW16 [32,33,34,41]. The position of RW16 in the membrane is not surprising, given its primary sequence and amphipathicity [42]. As already suggested for penetratin, the Arg residues in RW16 are mostly located at the lipid phosphate groups and form hydrogen bonds and salt bridges to act as an anchor in the membrane for the peptide [43]. Moreover, it was shown with MD simulations that polar side chains can establish long-term contacts with lipids by forming salt bridges and hydrogen bonds, and create local membrane perturbations [44]. Although this effect appears stronger with anionic membranes, where clustering of negatively-charged lipids occurred, it was also observed with zwitterionic membranes.

In our study, we noticed that some Arg residues are inserted deeper into the bilayer and locate at the glycerol region of lipids. We calculated the hydrogen bond number and confirmed that Arg residues establish long-term and stable hydrogen bonds with lipids and water molecules. Although membrane insertion of arginine is energetically unfavorable and associated with a high free energy cost, it has also been described that Arg residues can pull down water molecules in the membrane to stabilize its insertion. This process, known as water defect, was observed for the TAT peptide in zwitterionic membranes by neutron scattering experiments, and was shown to produce local membrane perturbations [45]. Different studies by all-atom MD simulations have indicated that this induces substantial membrane deformations due to the insertion of polar side chains in the hydrophobic core of the membrane [46,47,48,49]. This was further suggested as a mechanism for translocation of Arg-rich peptides [50] and might partially explain the membrane perturbation and leakage observed of RW16 in the presence of zwitterionic lipids. Despite strong Arg–water hydrogen bonds observed in our calculations, water insertion was not observed in our simulations, which at 1 μs was unable to be explored for the longer timescales (seconds/microseconds) required for this process. These observations would instead require enhanced sampling techniques, such as umbrella sampling. However, we did observe bidentate hydrogen bonding involving Arg residues with the lipid phosphate groups, and such interactions were reported to be important in cellular uptake mechanisms by creating a small curvature of the membrane, which could induce invagination phenomena [51,52]. These curvatures could contribute to tubulation and internal vesicle formation as induced by RW16 on giant unilamellar vesicles (GUVs) observed in Lamazière et al. [17].

Aside from this process, we have shown that Trp residues create hydrophobic contacts with the lipid acyl chains and therefore stabilize the peptide helix in the membrane hydrophobic core (Figure 8). Strong hydrophobic contacts with the lipid acyl chains were similarly observed using CD and NMR by Czajlik et al. [53].

Our results show that membrane-bound RW16 forms a stable α-helix with limited dynamics restricted to one terminus. The orientation of the helix appears to differ between NMR spectroscopy and MD simulations, and this difference might be explained by an altered helix polarity driven by the nature and size of the N-terminal cap. In NMR spectroscopy, the N-terminal biotin may prevent this end of the helix inserting into the membrane, while in MD simulations the acetyl group does not strongly influence the insertion of the peptide. Moreover, the membrane model used in both methods differs, and it is possible that the membrane curvature might affect the orientation of membrane-active peptides relative to the bilayer [20]. On one side, NMR measurements were performed in DPC micelles, which allows for fast molecular rotation as required for liquid-state NMR spectroscopy. On the other side, MD simulations used DOPC lipids which assemble in a fully flat membrane. The size and curvature of the membranes are therefore different and likely have an impact on how the peptide inserts in the membrane. Our data nevertheless converges on a tilted configuration of the peptide in zwitterionic bilayers (Figure 8).

Moreover, data obtained by NMR spectroscopy and MD simulations both reveal side chain contacts between Arg and Trp residues and, more specifically, π-cation interactions between Arg15 and Trp10, Trp11, and Trp14. A preference was observed between Arg15 and Trp11, which corresponds to the (i, i + 4) positions of the helix, i.e., to one turn of the α-helix. Such side chain contacts, in both buried and solvent-exposed positions, were shown to highly contribute to the conformational stability and the function of biomolecules [54,55]. Herein, a hydrophobic pocket surrounding Arg15 was formed by three tryptophans that help mask the positive charges of the Arg residue. Such pairing of aromatic and polar residues was indicated to decrease the energetic barrier for the motion of cationic side chains through a lower dielectric environment like the bilayer [44]. Similar π-cation interactions were also observed for penetratin between Arg and multiple Trp residues [32,33]. In our case, this process might help RW16 to further stabilize inside the bilayer, and hence create small fluctuations of the lipid membrane. These fluctuations could explain the strong and quick membrane leakage observed previously with zwitterionic liposomes in comparison to anionic liposomes [14].

Overall, the data obtained in this study clarify the membrane interaction and insertion properties of RW16 and connect these findings to observed perturbation of zwitterionic lipids. The stable conformational and insertion behavior observed for RW16 influences its activity toward biological membranes (i.e., cell internalization, antibacterial and antitumoral properties), and this was reported as an important property of CPPs like penetratin. Regardless of the peptide orientation in membranes, we obtained converging results (degree of insertion, tilting, side chain contacts) between the different methods employed in this study and comparable to similar studies on other CPPs [34]. Moreover, by using complementary approaches, we have shown that the choice of cap when protecting the terminal side of peptides may have functional importance and could impact peptide physico-chemical properties and membrane interaction.

## 4. Materials and Methods

### 4.1. Materials

All lipids were purchased from Avanti Polar Lipids (Alabaster, AL, USA). Gd(DTPA-BMA) was purified from the commercially available MRI contrast reagent Omniscan (GE Healthcare SAS, Vélizy-Villacoublay, France) by high performance liquid chromatography (HPLC). Omniscan was diluted with water to 250 mM and purified with an Alliance 2695 (Waters, Milford, MA, USA). The isocratic separation was performed on a semi-preparative C18 reversed phase (RP) column (Symmetry300RP-18, 300 mm × 10 mm, particle size 5 µm, Waters, Germany). The mobile phase was 100% water at a flow rate of 3 mL/min. Detection was performed with a variable wavelength detector set at 200 nm. Injection volume was 50 µL. Gd (DTPA-BMA) was eluted as a single peak (tR 9 min). The fractions were combined and lyophilized to obtain a white amorphous powder.

### 4.2. Liposome Preparation

All liposomes were prepared by initially dissolving the appropriate quantity of phospholipids in chloroform and methanol to ensure the complete mixing of the components and to obtain the desired concentration. A lipid film was then formed by removing the solvent using a stream of N_2_ (g) followed by 3 h vacuum. To form MLVs, the dried lipids were dispersed in Tris buffer 10 mM, 150 mM NaCl, 2 mM EDTA, and thoroughly vortexed. To form LUVs, the MLVs dispersion was run through five freeze/thaw cycles and passed 11 times through a mini-extruder equipped with a polycarbonate membrane with a pore diameter of 0.1 μm (Avanti Polar Lipids, Alabaster, AL, USA).

### 4.3. Molecular Dynamics Simulations

All-atom molecular dynamics (MD) of RW16 within a DOPC membrane were performed in this work. In order to generate the starting conformation for this system, we used the following strategy. First, an all-atom pure DOPC bilayer without peptide was pre-equilibrated for 200 ns. To get an idea of where the peptide partitions in the bilayer, we performed some self-assembly simulations with the MARTINI coarse-grained (CG) force field [56,57]. We then picked the NMR structure with the lowest energy and placed it in the (all-atom) bilayer using a typical CG snapshot as a template. This CG snapshot allowed us to place the NMR structure of RW16 at a probable vertical position, roughly with the center of mass of the peptide between glycerols and phosphates functional groups, and also with a relevant orientation so that Trp residues faced the hydrophobic core of the membrane and Arg residues were oriented towards water. Lipids and water molecules overlapping with RW16 were removed. The number of lipids in the other leaflet was adjusted to get the same number of lipids in both leaflets. Next, lipids were repacked against the peptide using an NPT simulation (at 300 K and 1 atm) of 10 ns with position restraints (PR) on the peptide. At this point, this was our starting conformation.

The system consisted of one RW16 peptide, 100 DOPC lipids (50 per leaflet), 3400 water molecules, and a quantity of Na^+^/Cl^−^ ions so that the system was neutral and the final ion concentration was 150 mM (10 Na^+^ and 20 Cl^−^). The termini of the peptide were capped with an acetyl at the N-terminus and an amide at the C-terminus. The total number of atoms was 16,233 and the volume of the box was approximately 6.0 × 6.0 × 6.7 nm^3^. Using the starting conformation described above, we performed three different runs using different initial velocities. For each run, an equilibration of 100 ps was performed with PR on the peptide, followed by a 1000 ns production (for which the PR were fully released).

All MD simulations were performed using GROMACS 5 [58]. Except for the CG self-assembly simulations described above, the OPLS-AA force field [59] was used for the protein in combination [60] with the Berger lipids for DOPC [61] and the TIP3P model [62] for water. Because OPLS-AA and Berger lipids have different 1–4 combination rules, the half-ε double-pairlist strategy was used to mix both force fields [60,63]. Electrostatic interactions were calculated with the particle-mesh-Ewald (PME) method [64], with a real-space cutoff of 1 nm. Bond lengths were constrained using the LINCS algorithm [65]. The integration time step was set to 2 fs. Water molecules were kept rigid with the SETTLE algorithm [66]. The system was coupled to a Bussi thermostat [67] and to a semi-isotropic Parrinello–Rahman barostat [68] at a temperature of 300 K and a pressure of 1 atm.

All analyses were performed using GROMACS tools. For each analysis on which we computed averages, the first 10 ns of the production runs were systematically discarded. RW16 secondary structures were determined with the DSSP program [69,70], implemented in the GROMACS tool do_dssp. Molecular graphics were generated with Pymol [38].

### 4.4. Fluorescence Spectroscopy Experiments

#### 4.4.1. Brominated Lipid Quenching Experiments

Depth-dependent fluorescence quenching of tryptophan of RW16 was performed in LUVs composed of DOPC and either (6,7)-, (9,10)-, or (11,12)-BrPC (70:30 mol:mol). Fluorescence intensities in the absence of quencher (F_0_) were measured in DOPC vesicles. Spectra were recorded between 300 and 500 nm with an increment of 1 nm, an integration time of 0.1 s, and using an excitation wavelength of 280 nm. The P/L molar ratio was 1/50 and the final peptide concentration was 0.5 µM. Data were corrected for vesicle background. Depth-dependent fluorescence quenching profiles (DFQPs) were fitted to the experimental points in Matlab using the distribution analysis (DA) method [26,27] and the parallax method (PM) [21,28,29,30] with the following equations:

(1) $$\mathrm{DA}:\;\ln\frac{F_{0}}{F_{\left(h\right)}}=\frac{S}{\sigma \sqrt{2\pi }}e^{\left[-\frac{{\left(h-h_{m}\right)}^{2}}{2\sigma^{2}}\right]}$$

(2) $$\mathrm{PM}:\;\ln\frac{F_{0}}{F_{\left(h\right)}}=\pi C\left[R_{c}^{2}-{\left(h-h_{m}\right)}^{2}\right]$$

where F_0_ is the Trp fluorescence intensity in absence of quencher, F_(h)_ is the Trp fluorescence intensity in the presence of quencher at the distance h(Å) from the bilayer center, and h_m_ corresponds to the average insertion depth of the tryptophan residues. In DA, the DFQP data are fitted with a Gaussian function where σ represents the dispersion, which is related to the in-depth distribution of the tryptophan chromophores, and S is the area under the quenching profile, which is related to the quenching ability of the tryptophan part. The parallax method fits data to a truncated parabola, and R_c_ is the radius of quenching. Average bromine distances from the bilayer center (h) for (6,7)-BrPC, (9,10)-BrPC and (11,12)-BrPC were taken to be 11.0, 8.3, and 6.5 Å, respectively [21].

#### 4.4.2. Acrylamide Quenching

Following peptide–lipid interactions, the accessibility of the peptides to aqueous quenchers of Trp fluorescence was modified. We used acrylamide as a Trp fluorescence quencher from a stock solution of 4 M. Acrylamide quenching experiments were performed with a 0.5 µM peptide solution in the absence or presence of LUVs and with a titration of acrylamide. The peptide/liposomes mixtures (1:50 mol:mol) were incubated for 15 min at room temperature prior to the measurements. The excitation wavelength was set to 295 nm instead of 280 nm to reduce the absorbance by acrylamide (ε ^280^ = 4.3 M^−1^·cm^−1^, ε ^295^ = 0.24 M^−1^·cm^−1^). Fluorescence intensities were then measured after the addition of acrylamide at room temperature. Data were analyzed according to the Stern–Volmer equation for collisional quenching [71]:

(3) $$\frac{F_{0}}{F}=1+K_{\mathrm{SV}}\cdot \left[Q\right]$$

where F_0_ and F correspond to the maximum fluorescence intensities in the absence and presence of quencher respectively, [Q] is the molar concentration of quencher, and Ksv is the Stern–Volmer quenching constant.

### 4.5. Circular Dichroism

CD spectra were recorded on a Jasco J-815 CD spectrophotometer with a 1 mm cuvette path length. Far-UV spectra were recorded from 180 to 270 nm with a 0.5 nm step resolution and a 2 nm bandwidth at 37 °C. The scan speed was 50 nm/min (0.5 s response time) and the spectra were averaged over 8 scans. CD spectra were collected for all the peptides in phosphate buffer at pH 5.5 with and without micelles at peptide/lipid (P/L) ratio of 1:50 (mol:mol). For each sample, the background (buffer) was automatically subtracted from the signal. Spectra were smoothed using a Savitzky–Golay smoothing filter and were deconvoluted to estimate the secondary structure content using the deconvolution software CDFriend developed in our laboratory (S. Buchoux, not published) [72].

### 4.6. NMR Spectroscopy Experiments

The RW16 peptide sample was prepared at a concentration of 1 mM in 400 μL of H_2_O/D_2_O (90:10, *v:v*) containing 50 mM sodium phosphate buffer at pH 5.5, and 60 mM of DPC-d_38_ forming zwitterionic micelles. NMR experiments were recorded at 310 K on a Bruker 800 MHz Avance III spectrometer equipped with a TCI ^1^H/^13^C/^15^N cryoprobe, or a Bruker 700 MHz spectrometer with a standard TXI triple resonance gradient probe.

For assignment, we used a 2D ^1^H,^1^H-TOCSY with a mixing time of 40 ms, collected with 40 scans for each of the 256 increments. Additional assignment and restraints for structure calculation were obtained from a ^1^H,^1^H-NOESY spectra with a mixing time of 150 ms, collected with 32 scans for each of the 228 increments. The solvent signal in both experiments was suppressed using two excitation sculpting blocks before the start of the acquisition. Partial assignment of ^13^C chemical shifts was accomplished with an ^1^H,^13^C-HSQC for which 352 scans were acquired for each of the 256 increments.

To measure solvent accessibility by paramagnetic relaxation enhancements, the RW16-DPC-d_38_ sample was titrated with Gd(DTPA-BMA) to final concentrations of 1, 2, 3, 4, 5, 7.5, and 10 mM. Proton T2 relaxation was estimated by crosspeak intensity as a proxy for T2 relaxation in the 2D ^1^H,^1^H-TOCSY and ^1^H,^1^H-NOESY spectra. All spectra were processed using Bruker Topspin 3.2 and analyzed by Sparky.

## Figures and Tables

**Table 1:** Stern–Volmer coefficients (K_SV_) determined by fluorescence quenching by acrylamide and normalized accessibility factors (NAF). The experiment was performed in duplicate.

	K_SV_ (M^−1^)		NAF	
	Buffer	DOPC	Buffer	DOPC
Penetratin	14.2 ± 0.3	7.5 ± 0.2	1	0.53 ± 0.02
RW16	16.6 ± 0.7	3.0 ± 0.2	1	0.18 ± 0.02

**Table 2:** NMR and refinement statistics for RW16. PDB entry: 6RQS.

**Distance and Dihedral Restraints**	
**Total Distance Restraints**	408
Intraresidue	171
Sequential (|i − j| = 1)	44
Short Range (1 < |i − j| < 5)	20
Long Range (|i − j| > 4)	9
Ambiguous	162
Dihedral Restraints	28
Structure Statistics	
Violations (mean and SD)	
Distance Constraints (Å)	0.020 ± 0.003
Dihedral Angle Constraints (°)	1.1 ± 0.3
Deviations from Idealized Geometry	
Bond Lengths (Å)	0.001 ± 0.000
Bond Angles (°)	0.306 ± 0.008
Impropers (°)	0.21 ± 0.01
Ramachandran plot (%) ^a^	
Most favored	86.7
Additionally favored	13.3
Generally allowed	0.0
Disallowed	0.0
Average pairwise rmsd (Å)	
Protein backbone all	0.5 ± 0.1
Protein heavy all	1.8 ± 0.2

^a^ Determined by using PROCHECK-NMR [23].

**Table 3:** Secondary structure percentages of RW16 calculated from CD spectroscopy in phosphate buffer alone or in the presence of DPC micelles or DOPC LUVs at a P/L ratio 1/50.

	Random Coil	α-Helix	β-Sheet
Buffer	54	46	0
DPC micelles	24	76	0
DOPC LUVs ^1^	40	60	0

^1^ Data from [14].

**Table 4:** Average insertion depths and fitting parameters of RW16 in DOPC LUVs (P/L 1:50 mol:mol) determined by the distribution analysis and the parallax method.

	Distribution Analysis (DA)			Parallax Method (PM)	
	h_m_ (Å)	δ (Å)	*S*	h_m_ (Å)	Rc (Å)
RW16	12.4	8.9	1.7	12.5	13.8

## Supplementary Materials

Supplementary materials can be found at https://www.mdpi.com/1422-0067/20/18/4441/s1.

## Abbreviations

CPP	Cell-penetrating peptides
AMP	Antimicrobial peptides
MAP	Membrane-active peptide
SAR	Structure–activity relationship
GUV	Giant unilamellar vesicles
NMR	Nuclear magnetic resonance
MD	Molecular dynamics
LUV	Large unilamellar vesicle
DOPC	Dioleoylphosphatidylcholine
DPC-d_38_	Dodecylphosphocholine-d_38_
HSQC	Heteronuclear single quantum coherence
TOCSY	Total correlation spectroscopy
NOE	Nuclear Overhauser effect
NOESY	Nuclear Overhauser effect spectroscopy
CD	Circular dichroism
