# Supplementary material for: Biophysical Insight on the Membrane Insertion of an Arginine-Rich Cell-Penetrating Peptide
Source: Int J Mol Sci. 2019 Sep 9;20(18):4441. doi: 10.3390/ijms20184441 (PMC6769507; doi:10.3390/ijms20184441)
Supplement: Supplementary file 1 [file ijms-20-04441-s001.zip › Supp_Files_proof/ijms-574485-supp_proofMLJ.docx]

Supplementary material

**Biophysical Insight on the Membrane Insertion of an Arginine-Rich Cell-Penetrating Peptide**

**Marie-Lise Jobin ^1,2,^*, LydieVamparys ^3^, Romain Deniau ^3^, Axelle Grélard ^1^,**

**Cameron D. Mackereth ^4^, Patrick F.J. Fuchs ^5,6^ and Isabel D. Alves ^1,^***

^1^ Institute of Chemistry & Biology of Membranes & Nanoobjects (CBMN), CNRS UMR5248, University of Bordeaux, Bordeaux INP, allée Geoffroy St-Hilaire, 33600 Pessac, France

^2^ Present address: Interdisciplinary Institute for Neuroscience (IINS), CNRS UMR5297, University of Bordeaux, 33000 Bordeaux, France.

^3^ University of Paris, Institut Jacques Monod, CNRS, 75013 Paris, France

^4^ INSERM U1212, CNRS UMR5320, ARNA Laboratory, University of Bordeaux, IECB, 2 rue Robert Escarpit, 33600 Pessac, France

^5^ Sorbonne University, École normale supérieure, PSL University, CNRS, Laboratoire des biomolécules (LBM), 75005 Paris, France

^6^ University of Paris, UFR Sciences du Vivant, 75013 Paris, France

***** Correspondence: [marie-lise.jobin@u-bordeaux.fr](mailto:marie-lise.jobin@u-bordeaux.fr) (M.-L. J.); [i.alves@cbmn.u-bordeaux.fr](mailto:i.alves@cbmn.u-bordeaux.fr) (I.D.A.); Tel.: +33-5-3351-4735 (M.-L. J.); Tel.: + 33-5-4000-6849 (I.D.A.)


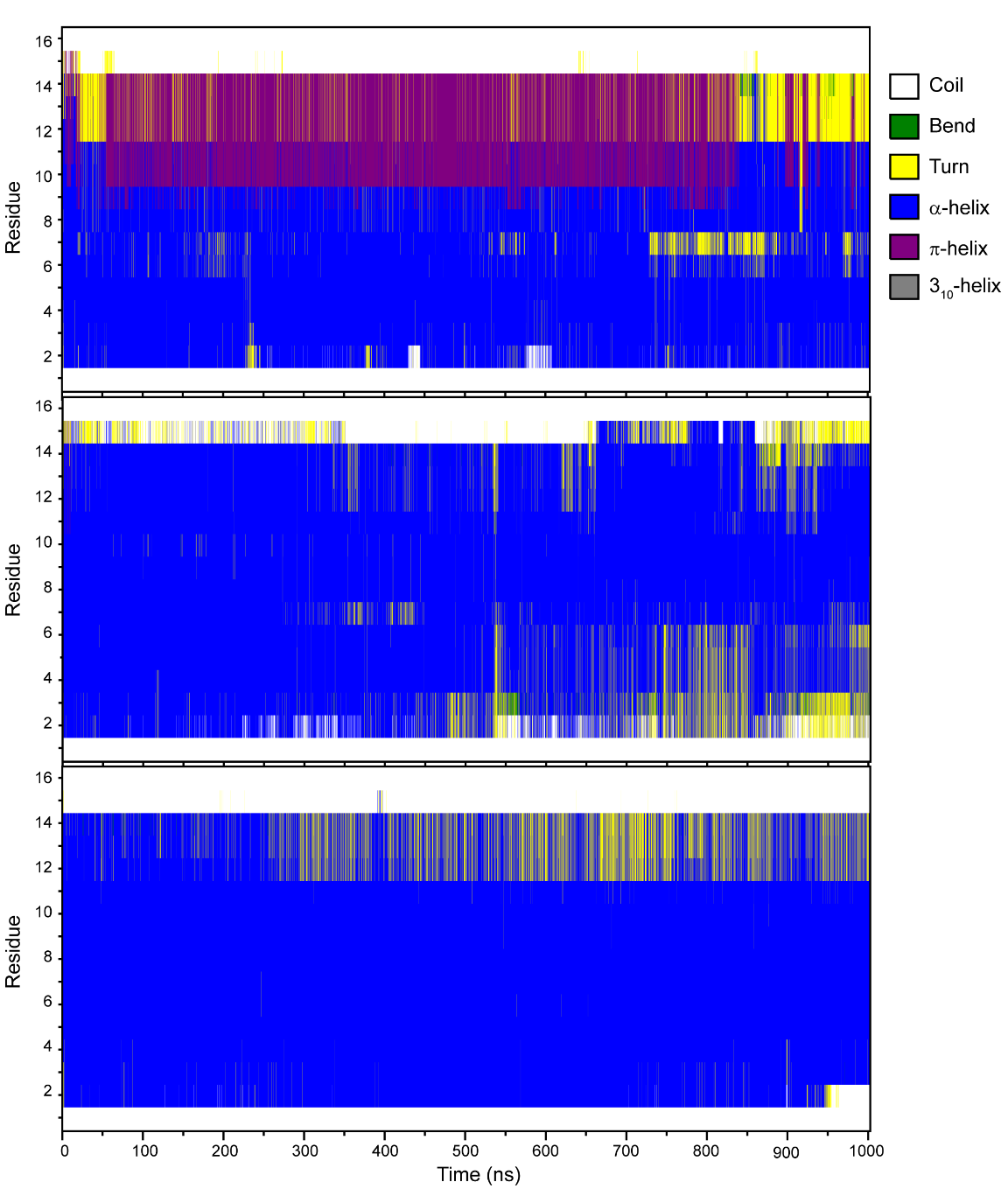


**Figure S1.** **Secondary structure of RW16 in the three MD trajectories** (top: trajectory 1; middle: trajectory 2; bottom: trajectory 3).


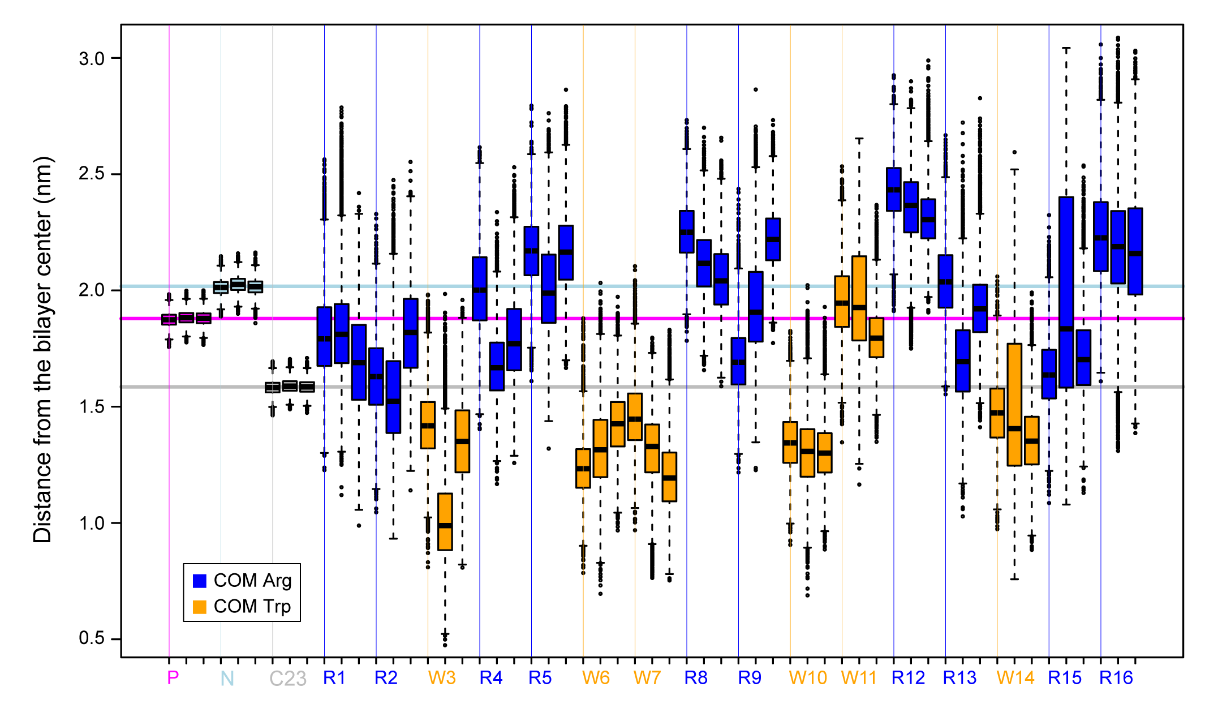


**Figure S2.** **Exposure of the peptide side chains determined by MD simulations.** Box plot of the vertical (*z*) position of the COM of the peptide side chains in comparison to the phosphorus of the lipid phosphate (P), the nitrogen of the choline (N) and the central glycerol atom (C23). Horizontal thick solid lines represent the average *z* position of P (magenta), N (cyan) and C23 (grey) atoms over the 3 trajectories. For each lipid atom / COM side chain, the 3 trajectories are shown (thin solid vertical line correspond to the first trajectory). Arg side chains are in blue, Trp side chains in orange. Large boxes (e.g. side chain of Arg15 of simulation 2) indicate that the side chain underwent an important change of partitioning within the bilayer in the corresponding simulation.


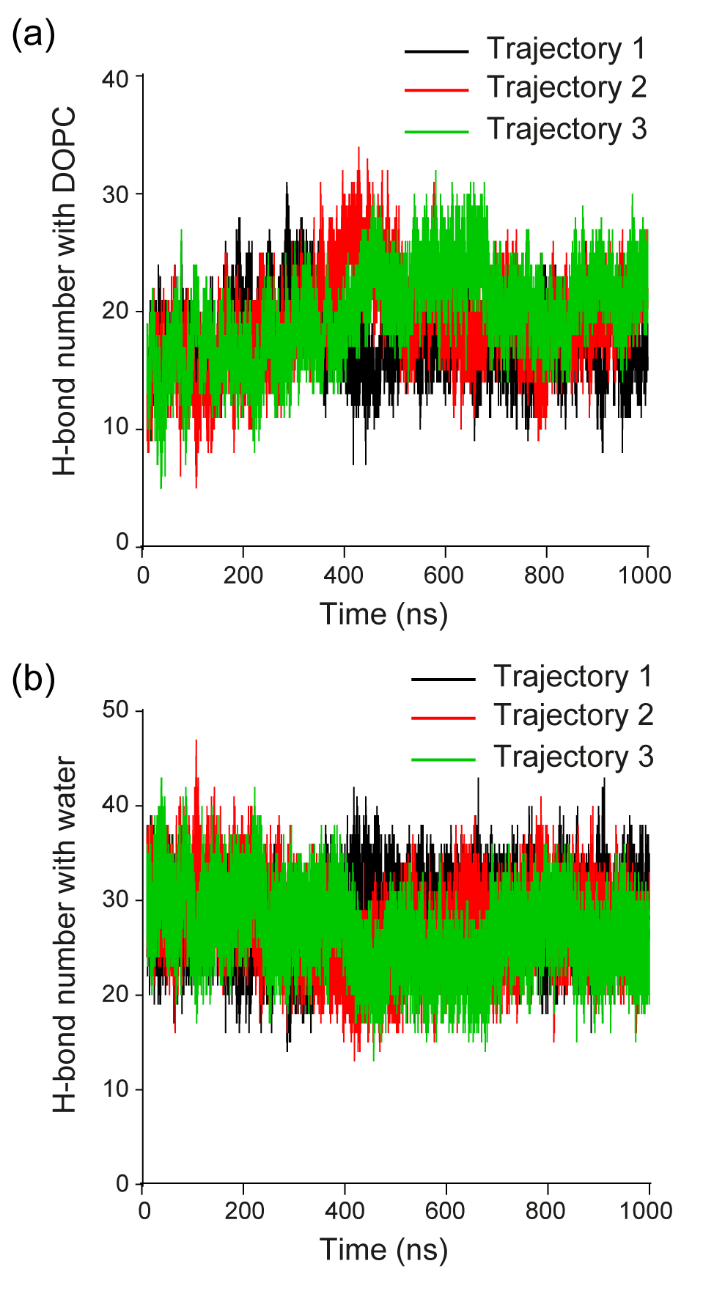


**Figure S3. Number of hydrogen bonds of Arg side chains of RW16 with lipids and water. (a, b)** Number of hydrogen bonds of Arg side chains with the carboxyl/phosphate groups of DOPC lipids (**a**) or molecules of water (**b**) determined over 1000 ns from three MD simulations.


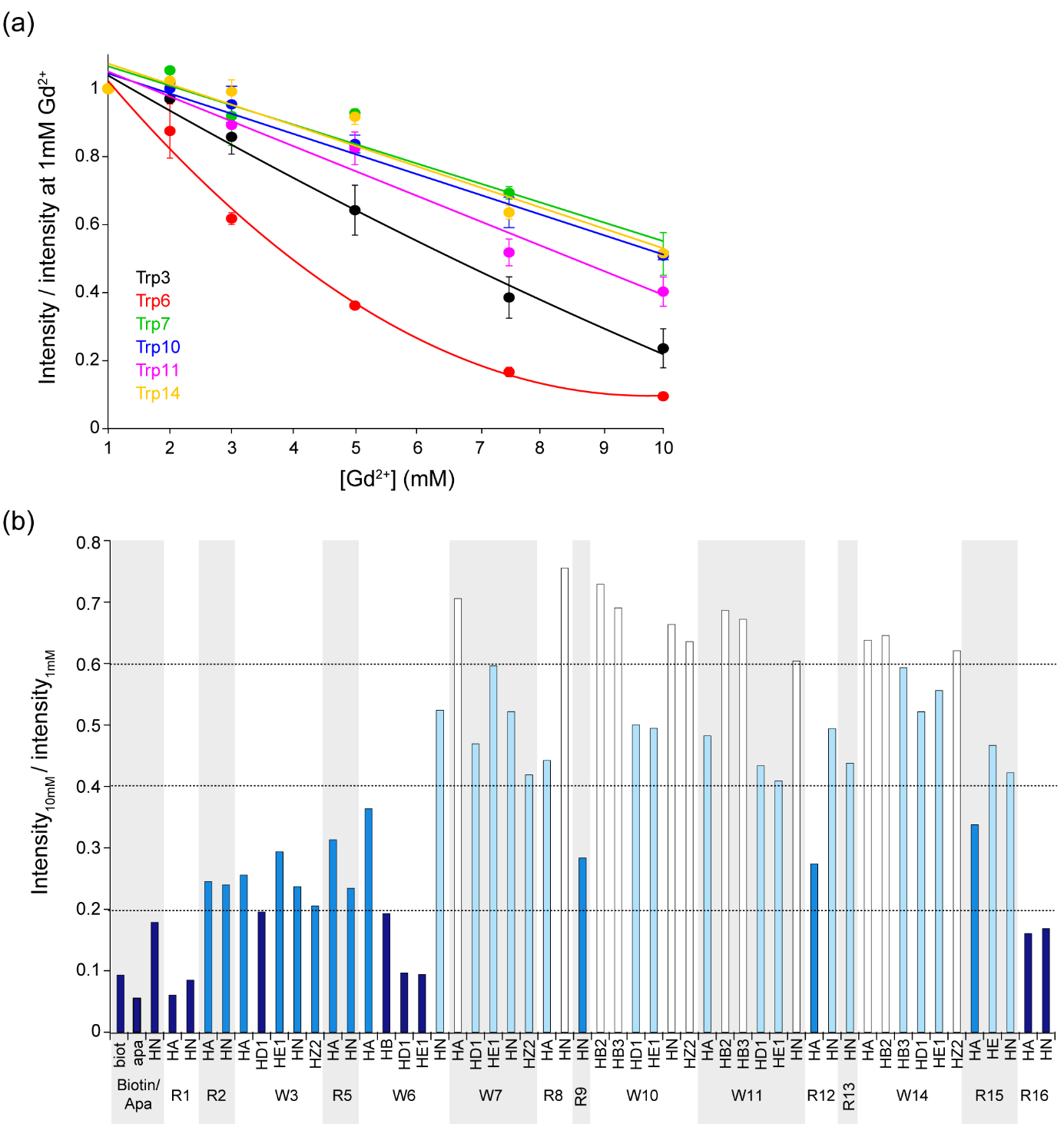


**Figure S4. Determination of peptide tilt by NMR spectroscopy. (a)** Addition of the paramagnetic compound Gd(DTPA-BMA) to the sample of RW16 in DPC-d38 micelles, at concentrations of 1, 2, 3, 5, 7.5 and 10 mM, on the Trp ^1^H^ε1^ nuclei. The paramagnetic relaxation enhancement uses the height of the crosspeak as a proxy for T2 relaxation. (**b**) A ratio of the crosspeak intensity at 10 mM versus 1 mM Gd(DTPA-BMA) provides a simple probe for solvent accessibility for several atoms in RW16. Nuclei that are closer to the solvent are more affected by Gd(DTPA-BMA) and thus have lower ratio values in this analysis.

**Supplementary Movie 1. Representative MD simulation of RW16 interacting with a DOPC bilayer**. The N-ter is on the left and sidechains are depicted in licorice representation with the Trp in orange and Arg in blue, the backbone is represented as a magenta ribbon and lipids are drawn as lines with the acyl chains in cyan and the Van der Waals spheres corresponding to the phosphorous.
