# Supplementary figures and images for: Biophysical Insight on the Membrane Insertion of an Arginine-Rich Cell-Penetrating Peptide
Source: Int J Mol Sci. 2019 Sep 9;20(18):4441. doi: 10.3390/ijms20184441 (PMC6769507; doi:10.3390/ijms20184441)

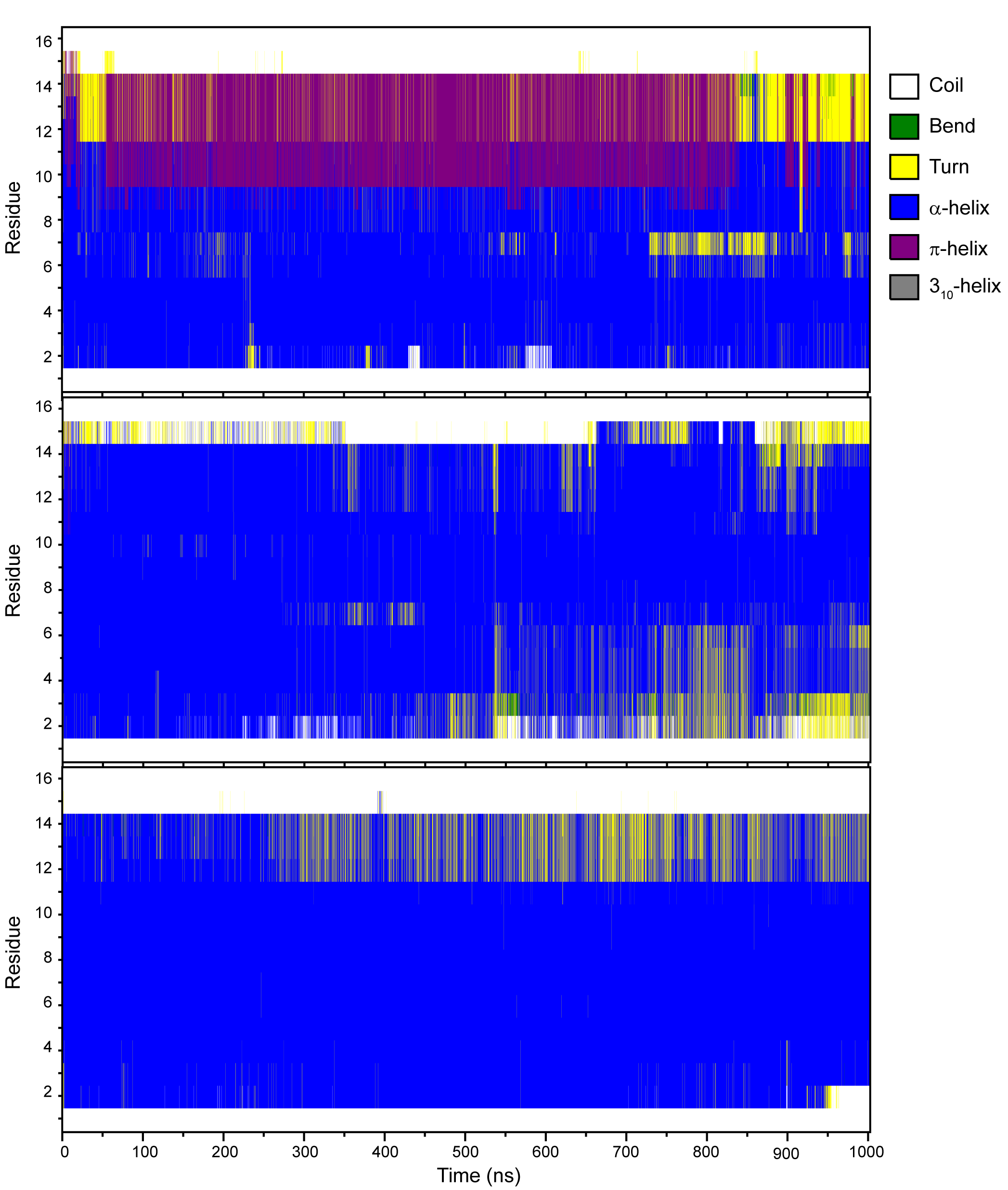

Supplement: Supplementary file 1 [file ijms-20-04441-s001.zip › Supp_Files_proof/FigS1.tif]

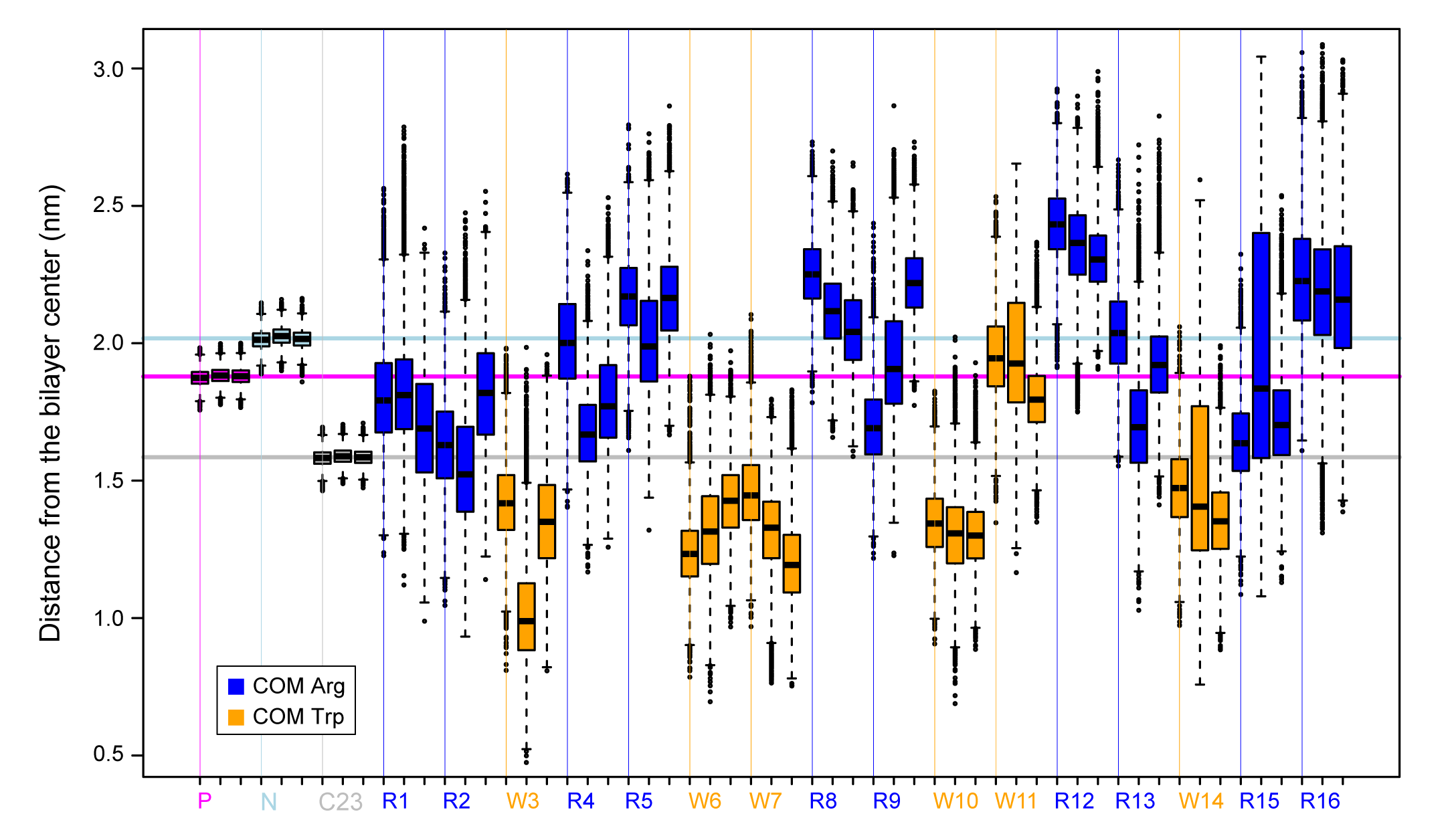

Supplement: Supplementary file 1 [file ijms-20-04441-s001.zip › Supp_Files_proof/FigS2.tif]

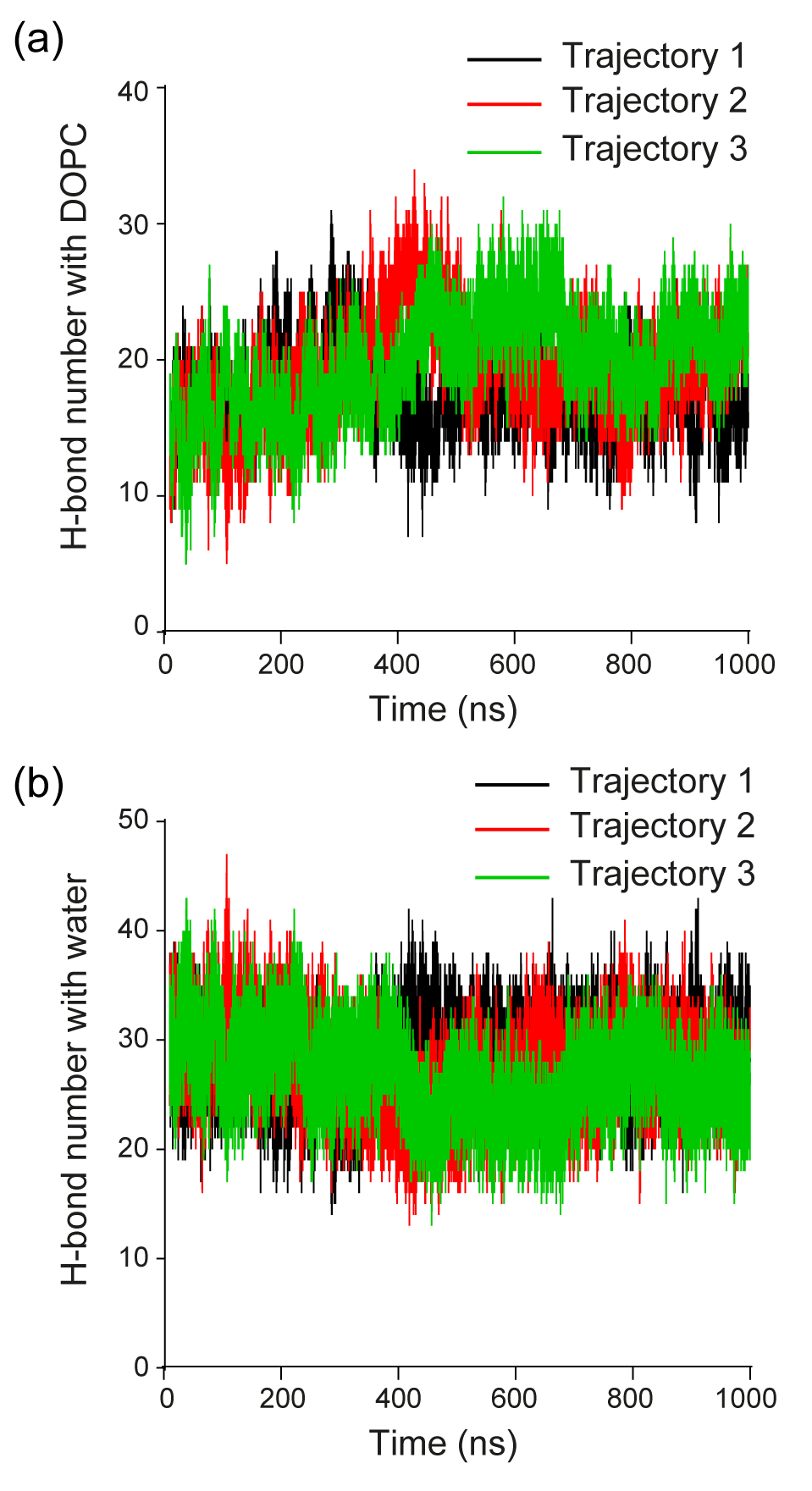

Supplement: Supplementary file 1 [file ijms-20-04441-s001.zip › Supp_Files_proof/FigS3.tif]

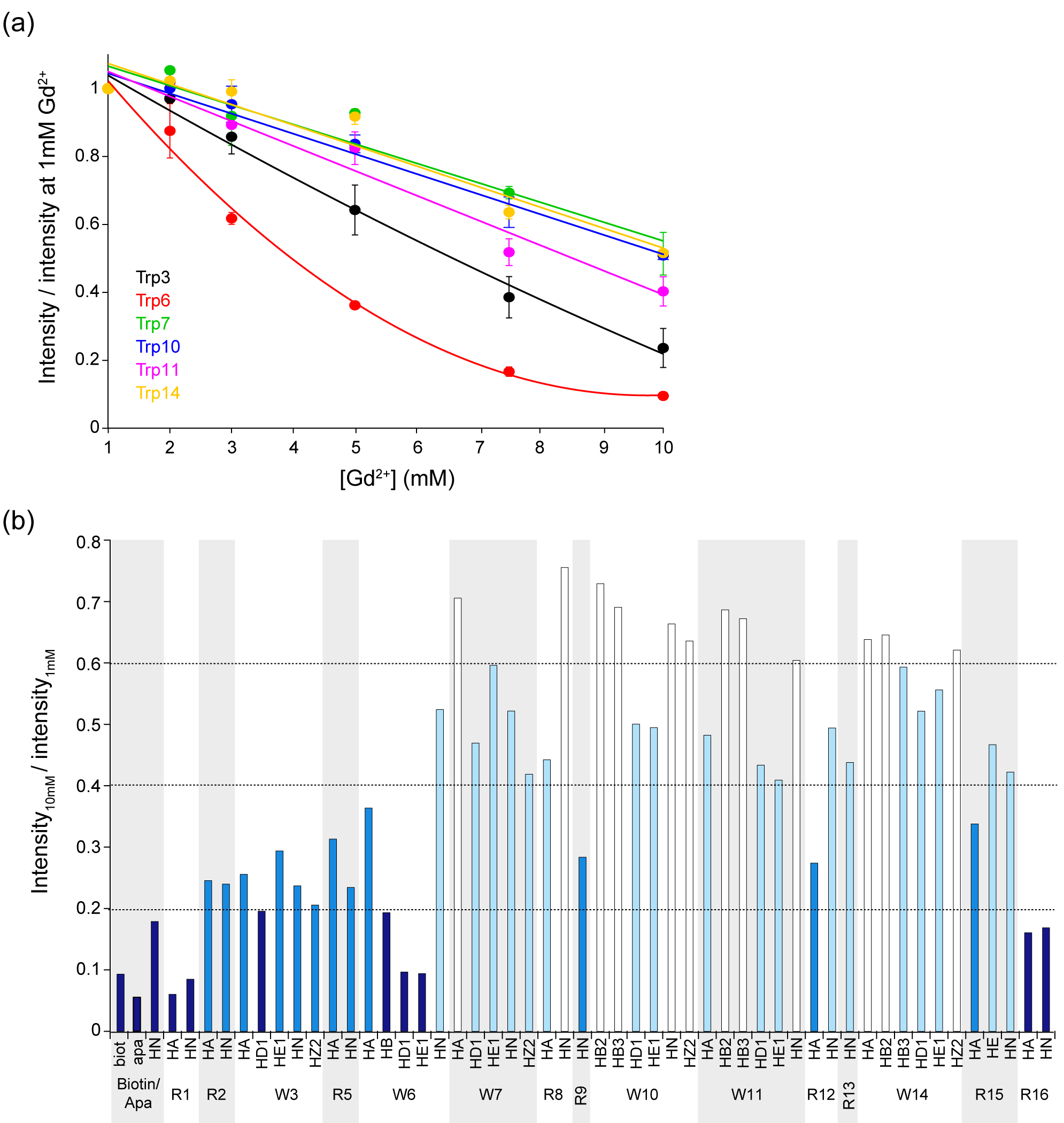

Supplement: Supplementary file 1 [file ijms-20-04441-s001.zip › Supp_Files_proof/FigS4.tif]
